# Vehicular Traffic Congestion Classification by Visual Features and Deep Learning Approaches: A Comparison

**DOI:** 10.3390/s19235213

**Published:** 2019-11-28

**Authors:** Donato Impedovo, Fabrizio Balducci, Vincenzo Dentamaro, Giuseppe Pirlo

**Affiliations:** Dipartimento di Informatica, Università degli Studi di Bari Aldo Moro, 70125 Bari, Italy; fabrizio.balducci@uniba.it (F.B.); vincenzo@gatech.edu (V.D.); giuseppe.pirlo@uniba.it (G.P.)

**Keywords:** vehicular traffic flow detection, vehicular traffic flow classification, vehicular traffic congestion, deep learning, video classification, deep learning, benchmark

## Abstract

Automatic traffic flow classification is useful to reveal road congestions and accidents. Nowadays, roads and highways are equipped with a huge amount of surveillance cameras, which can be used for real-time vehicle identification, and thus providing traffic flow estimation. This research provides a comparative analysis of state-of-the-art object detectors, visual features, and classification models useful to implement traffic state estimations. More specifically, three different object detectors are compared to identify vehicles. Four machine learning techniques are successively employed to explore five visual features for classification aims. These classic machine learning approaches are compared with the deep learning techniques. This research demonstrates that, when methods and resources are properly implemented and tested, results are very encouraging for both methods, but the deep learning method is the most accurately performing one reaching an accuracy of 99.9% for binary traffic state classification and 98.6% for multiclass classification.

## 1. Introduction

Urban roads and highways have, nowadays, plenty of surveillance cameras initially installed for various security reasons. Traffic videos coming from these cameras can be used to estimate the traffic state, to automatically identify congestions, accidents, and infractions, and thus helping the transport management to face critical aspects of the mobility. At the same time, this information can also be used to plan the mid and long-term roads mobility strategy. This is a clear example of a smart city application having also a strong impact on citizens’ security [1].

Studies dealing with traffic state estimation by videos adopt a common processing pipeline, which includes the following:

- The pre-processing of the video frames to highlight the useful elements (vehicles) and hide the unnecessary ones (background, etc.);
- The extraction of visual features able to describe the traffic state (e.g., number of vehicles, speed, etc.);
- One or more methods to classify the traffic state.

It is difficult to identify and select the best algorithms to be adopted because, often, systems reported in many studies are different at many stages, as well as adopt different datasets and testing conditions. This research provides a brief review of the most used techniques and reports an extended and systematic experimental comparison under common set-up conditions, thus highlighting strengths and weaknesses of each approach and supporting interested readers in the most profitable choices. More specifically:

- Haar Cascade, You Only Look Once (YOLO), the Single Shot MultiBox Detector (SSD), and Mask R-Convolutional Neural Networks (R-CNN) are adopted and compared for vehicle detection;
- Results provided by the two most accurately performing detectors, among the aforementioned, are used in a comparative schema to evaluate a set of visual features able to characterize the traffic state. These features were fed to four different classic classifiers, thus highlighting the most accurately performing one;
- Results obtained with classic approaches (previous point of this list) were compared to the use of deep learning techniques.

The article is organized as follows: Section 2 describes related studies, Section 3 presents the object detectors candidate to identify vehicles, the visual features useful to characterize a traffic video frame, and classifiers. The video datasets, evaluation metrics, along with experimental results are shown in Section 4. Section 5 presents conclusions and future researches.

## 2. Related Studies

Among the different methods that can be adopted to provide traffic flow (congestion) estimation, surveillance cameras play a crucial role. These systems can be installed without interfering with road infrastructures. Moreover, a large plethora of retrofit solutions are available in many cases, or systems have been already installed for some initial different aim. In any case, surveillance cameras can supply real-time information. The estimation of the traffic can be provided to users and police patrols to help in departures planning and congestion avoiding. Road panels or integrated vehicular monitors can also be used to reach the aim [2].

One of the first steps within the pipeline is vehicle identification [3]. Vehicles can be identified using features such as colors, pixels, and edges along with some machine learning algorithms. More specifically, detectors able to locate and classify objects in video frames exploiting visual features must be considered. State-of-the-art features are SURF (Speeded Up Robust Features) and bag-of-features [4], Haar Features [5], Edge Features [6], Shape Features [7], and Oriented Gradients Histograms [8]. Approaches based on visual features and machine learning models greatly benefited in efficiency with the introduction Convolutional Neural Networks (CNN) [9].

Choudhury et al. [10] proposed a system based on Dynamic Bayesian Network (DBN). It was tested on three videos after extracting visual features such as the number of moving vehicles [11]. Other examples of vehicle detector are in [12] and in [13] where features to characterize the traffic density are extracted from video footage. Li et al. [14] exploited the texture difference between congestion and unobstructed images, while similar approaches were used to recognize tow-away road signs from videos [15].

Vehicular traffic behavior can be also revealed by observable motion [16], in fact, it can be used to determine the number of vehicles performing the same action (e.g., by exploiting trajectory clustering for scene description). Drones and radio-controlled model aircrafts are exploited in the works of Liu et al. [17] and Gao et al. [18] to shoot live traffic flow videos and to study roads conditions. Shen and He [19] analyzed the vehicular behaviors at traffic bottlenecks and their movement to verify the decision-making process. In the research of Avinash et al. [20], Multiple Linear Regression (MLR) Technique was adopted to comprehend the factors influencing pedestrian safety. Koganti et al. [21] adopted Lane Distribution Factor (LDF) to describe the distribution of vehicular traffic across the roadway. Thomas et al. [22] presented a perceptual video summarization technique on a stack of videos to solve accident detection.

Real-time traffic videos have been used to observe temporal state of vehicles on a pedestrian crossing lane by using several image processing algorithms, connected components, and ray-casting technique [23]. Lin and Wang [24] implement a Vehicular Digital Video Recorder System able to support an online real-time navigator and an offline data viewer: The system was able to consider data security about vehicular parameters and to depict the instant status of vehicles.

## 3. Methods and Materials

Two approaches are compared in this research: The first one relies on visual features evaluated from traffic videos through computer vision algorithms using state-of-the-art object detectors and classifiers, the latter considers deep learning models able to automatically extract features from videos needed for the final classification.

### 3.1. Object Detectors 

Four different object detectors have been explored: Haar Cascade, You Only Look Once (YOLO), Single Shot MultiBox Detector (SSD), and Mask R-CNN.

The Haar Cascade object detector, originally developed by Viola and Jones [25], relies on a set of visual features able to exploit rectangular regions at a specific location identifying pixel intensities and differences between the regions. The term “Cascade” refers to the decision sequencing, in fact the algorithm constructs a “strong” classifier as a combination of weighted weak classifiers using a boosting technique. A search window moves through the whole image to cover all the pieces and, at the same time, it is resized to different scales to find objects of different sizes. The detector requires a set of positive and negative samples. Haar features are extracted in the test phase and then compared with those used in the training phase, in order to obtain a positive or negative identification. Haar Cascade has been successfully used for Vehicle Detection [26], also to evaluate a traffic congestion index [27].

The YOLO (You Only Look Once) object detector consists of a CNN called Darknet [28] with an architecture made by 24 convolutional layers working as feature extractors and 2 dense layers for the prediction. Successively, YOLO v2 introduced anchors as a set of boxes used to predict the bounding boxes, while in YOLO v3 the prediction is performed at different scales. The algorithm looks at the image only once and splits it into an NxN grid where each cell predicts a fixed number of bounding boxes to associate an object to the supposed class providing a confidence score. Many of the bounding boxes have a very low confidence. Therefore, they can be filtered by applying a threshold. YOLO is a fast algorithm because it requires only one image processing, at the same time accuracy decreases when two different objects are very close to each other. YOLO has been successfully used for Vehicle Detection in [29] where the focal loss is exploited and validated on the BIT-Vehicle dataset and in [30] where, on the same dataset, a mean Average Precision (mAP) of 94.78% is reported.

Convolutional Neural Network (CNN) is also at the basis of the *Single Shot MultiBox Detector* (SSD), producing a set of fixed-size bounding, a confidence score is provided representing the probability that the object in the box belongs to a specific class. The CNN is constituted by several layers that progressively decrease in size so that objects can be predicted on several scales. SSD has been successfully used for Vehicle Detection in [12] while Sreekumar et al. [31] developed a multi-algorithm method for the real-time traffic pattern generation and Asvadi et al. [32] exploited a Dense Reflection Map (DRM) inputted to a Deep Convolutional Neural Network for the vehicle detection.

Mask R-CNN is the evolution of R-CNNs [33]. The original R-CNN detector used selective search to extract a huge number of candidate (proposal) regions (2000) from the input image. The algorithm first generates many candidate regions by performing initial sub-segmentation. Then, a greedy approach combines similar (adjacent) regions in larger ones. The selective search and the greedy approach result in very low computing [34]. R-CNN developed by Microsoft Research addresses this speed computation problem. One single model is used to extract features from each region, predict the belonging class, and compute the box coordinates. This is performed on a filtered image, which uses the low rank approximation with Singular Value Decomposition (SVD) of the original image. Fast R-CNN is 10× faster than R-CNN. Faster R-CNN [34] improves Fast R-CNN by using an additional network, called Region Proposal Network, in place of the selective search (originally derived from R-CNN) used for the generation of the regions of interest. This increases the prediction speed by about 10× [35]. Mask R-CNN is built on top of Faster R-CNN and, in addition to Faster R-CNN, it provides also the object segmentation. The mask of the segmented object could be used for inferring the valid shape of the classified object.

It is important to note that, while SSD used pre-trained weights on the Pascal Visual Object Classes (VOC) dataset [36], YOLO v3 and Mask R-CNN used pre-trained weights on the Coco dataset [37].

### 3.2. Visual Features

The output produced by a specific detector can be exploited to evaluate the traffic state and its density. In this research, five visual descriptors were considered: Total Vehicles, Traffic Velocity, Traffic Flow, Road Occupancy, and the Texture Feature.

The *Total Vehicles* feature is the number of bounding boxes provided by the vehicle detector and evaluated for each video frame [10,13,20,21,24,38,39,40,41,42,43].

The Traffic Velocity (average speed of all vehicles in the frame) is evaluated by tracking the vehicular bounding boxes centers and calculating the Euclidean distance with the corresponding position in the next frame [31]. The distance of each vehicle has been normalized according to the fps (frame per second) rate thus finding the individual speed and the global average speed (the sum of all vehicles velocities divided by their number). The last parameter can be considered as an estimation of the global traffic velocity [12,13,42,44,45].

The third visual feature is *Traffic Flow*, it is calculated considering the difference between the incoming and the outgoing traffic, respectively evaluated as the number of vehicles entering the camera field of view and those leaving it [15,39,40,42,45].

**Figure 1:**
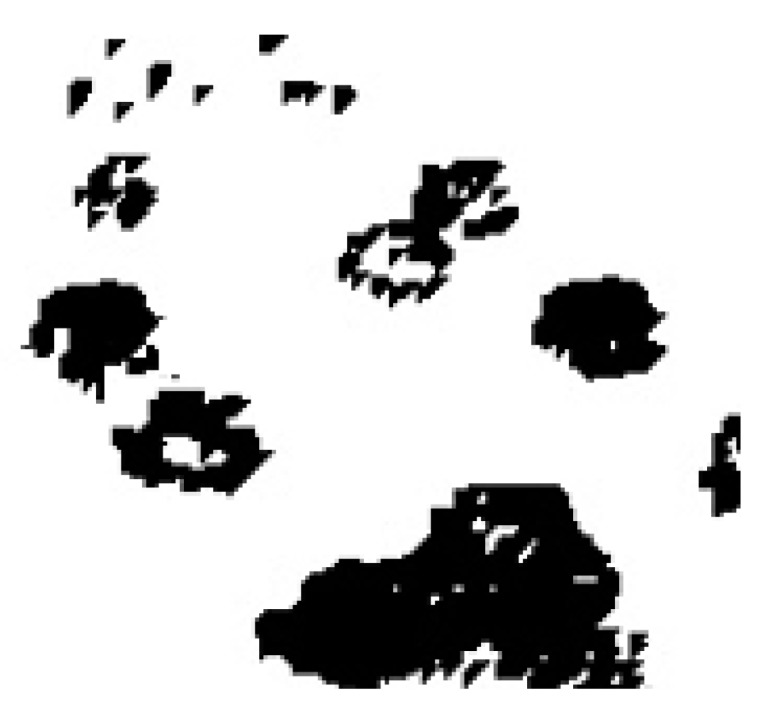
The morphological operator applied to the frame of the vehicular traffic video.

Background suppression and morphological transformations (i.e., opening and closing) can be used to isolate vehicle shapes: This process returns the *Road Occupancy* providing a relationship between the flat road surface (white pixels in Figure 1) and vehicles (black pixels in Figure 1). To gain this result, frames are converted to grayscale. Successively, pixel values are subtracted from the next frame highlighting non-modified areas (background) and modified ones (foreground): Pixels belonging to vehicles shape are those related to changes in the scene. A threshold operator (i.e., Regional Minimum) is adopted to distinguish flat areas from areas occupied by vehicles which result from the ratio between black and white pixels dynamically changing according to the number of vehicles [9,12,16,41,42,44,45].

The *Texture Feature* was calculated according to the Gray Level Co-occurrence Matrix (GLCM) method [14,45,46,47]. This parameter is typically used to estimate vehicles’ density by exploiting the corresponding ‘energy’ and ‘entropy’ values. More specifically, energy reveals whether the texture is thick (high value) or thin, while the entropy expresses how the elements are distributed (uniformly featuring a high value or not) [48]. The value of energy is inversely proportional with vehicle density, and the value of entropy is proportional with vehicle density. In other words, the gray histogram of image should be distributed uniformly and the texture of the image should be thin in case there are many vehicles in a frame. Therefore, the energy feature value should be small, and the entropy feature value should be big.

### 3.3. Machine Learning Classifiers

In this research three classifiers were considered and compared: k-Nearest Neighbors, Support Vector Machine, and Random Forest.

The K-Nearest Neighbors (KNN) is used for classification and regression tasks exploiting sample vectors in a multi-dimensional feature space. K is a user-defined parameter referred to the number of class labels: the unknown input vector is classified assigning it the “nearest class” among those already known. Many distance measures can be considered as, for example, the Euclidean or the Manhattan one. K-NN has been applied to this specific field in [38] and [49].

Support Vector Machine (SVM) maps feature vectors of two different classes within a hyperspace and searches for the best separating hyperplane taking into account only a reduced set of the initial amount of examples (called support vectors), which are those difficult to be classified. According to data distribution, different separating hyperplanes (kernel) can be considered. In this research the linear kernel and *a Gaussian Radial Basis* (rbf) function were considered as in [50] where the traffic congestion is classified through a comparison between AlexNet+SVM and VGGNet+SVM.

The Random Forest (RF) classifier relies on a bagging method to build several “base learners”, usually decision trees. The base learners are successively combined to provide the final classification. RF repeatedly selects a bootstrap sample from the training set. It selects a random subset of features, and then fits a decision tree to this sample. Due to this randomness, the bias of the forest increases, but, due to averaging, its variance also decreases. In extremely randomized trees (ET), randomness is taken a step further by also completely randomizing the cut-point choice while splitting each node of a tree. This allows the variance to be reduced a little more at the expense of a slight increase in bias. In this research, 100 decision trees were used as base learners for RF and ET.

### 3.4. Deep Learning Classification Models

He et al. [51] proposed a residual deep learning framework for image classification, where layers were redrafted in order to learn residual functions with respect to the input layer. The proposed ResNET had 34 layers that follow the same pattern while performing 3 × 3 convolutions with a fixed feature map dimension, the input is bypassed every two convolutions. Moreover, the width and height dimensions remain constant for the entire layer thus reducing the complexity per layer of the network. The output is a binary classification (congested/not congested). The ResNET has been also re-trained in [52] on the Shaanxi Province dataset.

Kurniawan et al. [53] used two convolutional layers, a max pooling layer, and a fully connected layer where the first two layers are convolute with 3 × 3 filters and 32 feature maps, the third one is a 2 × 2 max pooling layer used for down-sampling, and the last one is a fully connected layer with 128 neurons. Rectified Linear Units (ReLU) activation function has been exploited in both the convolutional and fully connected layers while a sigmoid activation function has been used for the output layer [45,50].

## 4. Experiments and Discussion

### 4.1. Video Datasets

Different datasets, used for different aims, were adopted in this research. The GRAM Road Traffic Monitoring (RTM) is generally used for vehicle detection and it was adopted here to evaluate and compare performance of object (vehicles) detectors [54]. Trafficdb contains annotations related to the state of the traffic and it is here used to compare classification techniques [55,56].

#### 4.1.1. GRAM RTM

The Road-Traffic Monitoring [54] is a dataset specifically used for vehicular detection in a traffic environment. It consists of three video sequences from which individual frames were labeled with bounding box around vehicles. The “M-30” video includes 7520 frames recorded on a sunny day with a Nikon Coolpix L20 camera having a resolution of 640 × 480 pixels at 30 fps. The second video “M-30-HD” includes 9390 frames recorded in the same place of the previous video but on a cloudy day at a higher resolution (1280 × 720 pixels at 30 fps using a Nikon DX3100 camera). The last “Urban1” video contains 23,435 frames in low resolution (480 × 320 pixels at 25 fps). This dataset offers the possibility to evaluate vehicles detectors under different working conditions.

**Figure 2:**
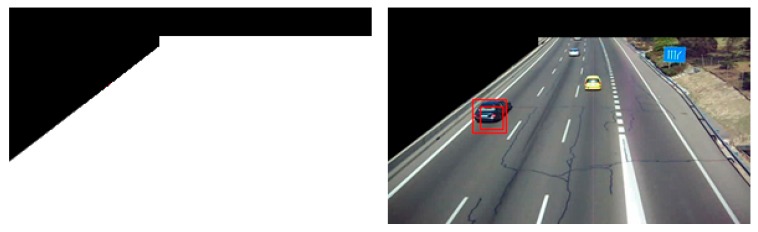
GRAM dataset: (**left**) a pixel mask that highlights the Region of Interest about vehicles present, annotated (and subsequently trackable) in each video frame (**right**).

From each video of the dataset, the ground-truth consists in bounding boxes around all vehicles per each frame. Information about the acquisition properties are provided, together with pixel masks useful to extract region of interests (ROI) and decrease the computational load of subsequent processing phases (Figure 2).

#### 4.1.2. Trafficdb

**Figure 3:**
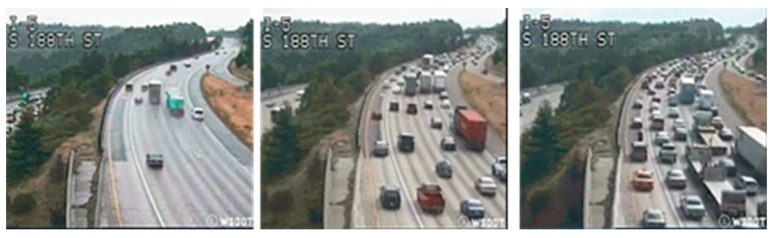
Three images from Trafficdb that depict a traffic state classified as Light, Medium, and Heavy.

Trafficdb dataset is a state-of-the-art dataset used for vehicular traffic state classification since it is provided with specific annotations [55,56]. It is constituted by 254 videos acquired between 8 April and 8 June 2004 on Seattle (USA) highway segments. The ground-truth includes three classes (Figure 3): ‘Heavy’—very congested traffic, ‘Medium’—low vehicular flow, and ‘Light’—normal travel velocity.

### 4.2. Evaluation Metrics

Vehicle bounding boxes provided by the object detector can be compared to the real vehicle annotation highlighting the most accurately performing one. It is quite clear that the result of this phase has a strong impact on all the subsequent stages within the processing pipeline.

The metric *Correct detections* refers to correctly detected vehicles for each frame and it is measured as the pixel intersection between the original ground-truth bounding box area (named G) and the predicted one (named P). The *Jaccard Index* or *Intersection over Union* (1) was considered.

(1) J(G,P)=|G∩P||G∪P|.

The second performance metric here considered is the *Computing time* evaluated by adding the processing time for each video frame at each iteration. It is useful to select the most suitable detector for a specific problem (real-time, on site, off-line, etc.).

The accuracy was considered and evaluated as follow:

accuracy = (TP + TN)/(TP + TN + FP + FN),(2)

where:

• TP: the true positive samples, example of class X and classified by the system as X;

• TN: the true negative samples, object not of class X and not classified by the system as X;

• FP: the false positive samples, object not of class X but classified by the system as X;

• FN: the false negative samples, object of class X, but not classified by the system as X;

Experiments were performed on a System featuring Ubuntu 18.04 as Operating System, AMD Ryzhen threadripper 1920x with 12 cores, Nvidia Titan RTX 24 GB RAM, 64 GB RAM DDR4.

### 4.3. Vehicle Detectors Evaluation

Table 1 reports results of the object detectors on the Road-Traffic Monitoring GRAM dataset.

The experimental phase pointed out that the Haar Cascade detector is the fastest one on each dataset. However, it provides good accuracy only on M-30-HD (75%).

The lowest processing time is achieved on the ‘Urban1’ video due to the low frame resolution that also impacts on the correctly identified vehicles, with 40% of accuracy also due to incorrect multiple detections.

The Single Shot MultiBox Detector (SSD) is the slowest object detector. The experiment on the "M-30" video reported an accuracy of 22%, which is the lowest between the four solutions. On the "M-30-HD" video, the computational time is much heavier (11 to 14 s) with frequent peaks between 14 and 17 s, the reported accuracy reaches 70%.

YOLO detector offers the best compromise with acceptable execution times and very good performances on all datasets.

Mask R-CNN exhibits very discordant results in terms of accuracy. In particular, the poor performance on Urban1 dataset is probably due to the poor image quality: JPEG compression kneaded colors, and thus cheated the Region Proposal Network.

The most accurately performing detector, considering the processing time, is Haar Cascade, while YOLO represents the compromise between time resources and detection accuracy.

### 4.4. Traffic State Classification: Visual Features and Machine Learning Classification

Results obtained in the previous section clearly report YOLO and Haar Cascade as the most accurately performing vehicles detectors in terms of accuracy and processing time. For this reason, they have been chosen to support the visual features extraction to build the input vector for vehicle classifiers.

The first experimental session involves the five visual features calculated using the best two selected object detectors with the different machine learning classifiers seen in Section 3.3. A 10-fold cross validation setup was adopted to minimize the effect of variance when choosing the training and testing examples. Classification results on the Trafficdb dataset are reported in Table 2. Visual features were extracted on a sampling rate of 30 frames.

Table 2 shows that YOLO detector combined with the Random Forest is the most accurately performing solution with an accuracy of 84%. The confusion matrix provided by this solution is reported in Table 3. Due to the unbalance of the classes in Trafficdb (the ‘Heavy’ class instances are about four times the other two), the classification results are provided in a normalized form.

### 4.5. Traffic State Classification: Deep Learning 

The deep learning models described in Section 3.4 were implemented and re-trained on the Trafficdb video dataset in a 10-fold cross validation setup. The ResNet [51] and the deep network architecture proposed in [52] were originally tested by respective authors on a two class (Heavy vs. Light) traffic state classification. To perform similar tests, at a first stage, samples labeled as ‘Medium’ were removed from the Trafficdb: results are shown in Table 4.

Finally, to compare results to the cases if the previous section, the two deep learning architectures were extended to perform traffic state classification on three classes. In this case, the best performance was reached by the ResNet [51] with an accuracy of 98.61% (results are in Table 5).

## 5. Conclusions

A pipeline to develop state-of-the-art traffic state classification systems from videos has been presented in this research. The pipeline is made up of three main steps: vehicle detection, feature extraction, and classification. Several state-of-the-art approaches have been considered and compared. A preliminary comparison between object detectors, performed on the GRAM Road Traffic Monitoring video dataset, has pointed out that YOLO v3 can be used for real-time vehicle detector exhibiting a detection accuracy of over 80%.

For the traffic state classification, two different approaches have been studied and tested on the Trafficdb video dataset. The first approach relies on visual features calculated through computer vision techniques and machine learning classifiers while the second one exploits deep learning able to embed the features extraction when training the model on the annotated dataset. In the classic approach (visual features and machine learning classifiers), the Random Forest has gained 84% of accuracy while the deep learning approach has reached an accuracy of over 98% with the same experimental setup, and thus showing a noticeable increase of +14% in the results.

The problem here considered is obviously complex, and the provided results need further improvement as for example: refinements of the object detection algorithms, validation on more traffic datasets. The last aspect is non-trivial because different road settings (country road, city road, road junction and crossroad, double lane roads, etc.) and weather conditions (rainy nights, fog, snow, gusts of wind, and so on) could have significant impact on systems.

## Figures and Tables

**Table 1:** Performance comparison according to the processing time and vehicle detection accuracy of the four object detectors on the three videos in the GRAM dataset.

		M-30	M-30-HD	Urban1
**Haar Cascade**	Time [s]	0.08–0.13	0.3–0.44	0.02–0.06
	Accuracy	43%	75%	40%
**SSD**	Time [s]	4–7	11–14	2.6–5.6
	Accuracy	22%	70%	69%
**YOLO v3**	Time [s]	1.0–1.8	1.0–1.8	1.0–1.8
	Accuracy	82%	86%	91%
**Mask R-CNN**	Time [s]	2.4–3.0	2.4–3.0	2.4–3.0
	Accuracy	89%	91%	46%

**Table 2:** Traffic state classification accuracy on the Trafficdb dataset.

	KNN	SVM (Linear)	SVM (rbf)	Random Forest
**YOLO v3**	0.81 ± 0.10	0.78 ± 0.12	0.79 ± 0.16	**0.84 ± 0.13**
**Haar Cascade**	0.66 ± 0.21	0.64 ± 0.11	0.64 ± 0.08	0.68 ± 0.21

**Table 3:** Normalized confusion matrix of the traffic state classification reached by the Random Forest classifier on the Trafficdb video dataset.

Random Forest	Light (Pred)	Medium (Pred)	Heavy (Pred)
**Light**	**0.94**	0.02	0.04
**Medium**	0.42	**0.47**	0.11
**Heavy**	0.20	0.11	**0.68**

**Table 4:** Normalized confusion matrix about the binary traffic state classification performed by the deep neural network of Kurniawan et al. [53] on the Trafficdb video dataset.

Deep Learning Architecture [44]	Light (Pred)	Heavy (Pred)
**Light**	**0.995**	0.004
**Heavy**	0	**1**

**Table 5:** Normalized confusion matrix about the multiclass traffic state classification performed by the deep neural network of Kurniawan et al. [53] on the Trafficdb video dataset.

Deep Learning Architecture [43]	Light (Pred)	Medium (Pred)	Heavy (Pred)
**Light**	**0.997**	0.003	0.
**Medium**	0.004	**0.972**	0.025
**Heavy**	0.	0.040	**0.959**

## Code

https://gitlab.com/islabuniba/traffic_benchmark.
